# Factors influencing the professional identity of medical care assistants: a qualitative study

**DOI:** 10.3389/fmed.2026.1956285

**Published:** 2026-10-16

**Authors:** Jing Zhao, Dongping Xu, Ying Hong, Hongjuan Chang, Yicheng Xiong

**Affiliations:** 1 Wuhan Asia General Hospital Affiliated to Wuhan University of Science and Technology, Wuhan, China; 2 School of Medicine, Wuhan University of Science and Technology, Wuhan, China; 3 Taixin Kanghu (Tianjin) Health Management Co., Ltd., Wuhan, China

**Keywords:** medical care assistants, professional identity, qualitative research, social identity theory, thematic analysis

## Abstract

**Objective:**

This study aimed to explore the factors influencing the professional identity of medical care assistants (MCAs) and to interpret relevant findings through the lens of social identity theory, thereby informing strategies to support professional identity and workforce stability.

**Methods:**

A qualitative descriptive research design was adopted. Using purposive sampling, 15 MCAs from a tertiary grade A hospital in Wuhan, Hubei province, China, were recruited for one-to-one semistructured interviews. Data were analyzed via inductive thematic analysis, and this report was prepared according to the Consolidated Criteria for Reporting Qualitative Research (COREQ).

**Results:**

Four themes and eight subthemes were identified: social evaluation influencing experiences of professional value and dignity, including recognition of professional value and challenges to professional dignity; role positioning and practical needs constructing professional meaning, including professional role positioning and practical needs as a driving factor; care interactions and self-adjustment shaping professional experiences, including communication and trust building and professional competence and emotional adjustment; and team and organizational support strengthening professional identity, including acceptance by the healthcare team and professional training and institutional support. Participants described social evaluation, professional role positioning, recognition or devaluation experienced during care interactions, team acceptance, and organizational support as factors influencing their professional identity. Practical needs primarily shaped occupational choice and intentions to remain, whereas self-adjustment functioned as a coping process within emotionally demanding care experiences.

**Conclusion:**

Accounts from participants suggest that the professional identity of MCAs is shaped by social evaluation, role positioning, care interactions, and team and organizational support within the context of their workplace. Healthcare institutions and relevant employers should enhance social and team recognition, clarify responsibilities of the role and collaboration boundaries, improve training and supportive employment arrangements, and consider performance-based incentives to support professional identity and workforce stability of MCAs.

## 1 Introduction

Medical care assistants (MCAs) are defined as personnel who provide daily care for patients and other individuals who have care needs in healthcare institutions and related care settings, and they perform certain supportive tasks under the guidance of healthcare professionals (1). With the increasing aging of the population and the growing care needs of patients with chronic diseases and functional impairments, MCAs have become increasingly important in assisting with patients' daily care, thereby reducing the burden of family caregiving and supporting clinical care (2). In recent years, China has successively introduced requirements for MCA training and standardized management, issued the National Occupational Standard for Medical Care Assistants, and implemented pilot programs for no-companion care services in hospitals, promoting the development of this profession toward greater standardization, professionalization, and hierarchical management (3).

Although the policy positioning and service demands of MCAs have become increasingly clear, their professional development may still be affected by factors such as insufficient social recognition, unclear role boundaries, high work pressure, limited compensation and incentives, and inadequate career development pathways. The MCA workforce commonly faces challenges related to insufficient occupational stability and relatively high turnover rates (4). These occupational environmental and developmental challenges not only affect work experiences of MCAs but may also influence their perception of their professional roles and occupational value, thereby affecting their professional identity (5, 6).

Professional identity refers to an individual's cognition; emotions; and self-positioning regarding their professional roles, professional values, occupational norms, and sense of belonging to the professional group. Unlike job satisfaction, which primarily reflects individual-based evaluations of their jobs, or organizational commitment, which concerns attachment to a particular organization, professional identity concerns how individuals understand and position themselves as members of a professional group. Professional self-worth may form part of this experience but does not encompass the broader dimensions of the professional role, group membership, and belonging. Moreover, it is closely associated with professional engagement, intentions to remain in the profession, and occupational stability (7, 8). Research in health professions has shown that professional identity encompasses multiple dimensions, including individual experiences, group belonging, learning and qualifications, and care contexts (5, 9). Studies among community nurses have further demonstrated that organizational support can influence burnout through professional self-concept (10). These findings suggest that professional identity is closely related to individuals' professional experiences and the social and organizational environments in which they work. However, existing research on professional identity has primarily focused on groups such as nurses, physicians, and nursing students (5), whereas studies on MCAs have mainly examined personnel training, standardized management, service capacity, and quality of care. Qualitative explorations of how MCAs understand their professional identity and the factors influencing their professional identity remain limited (11). MCAs differ from nurses in terms of professional entry requirements, scope of responsibilities, positioning within healthcare teams, and career development pathways (12, 13). Therefore, findings from existing studies on established health professions may not fully explain the professional identity of MCAs or the factors influencing its development.

Social identity theory (SIT) suggests that individuals define themselves as members of particular social groups through social categorization and incorporate group membership, along with its associated values and emotional meanings, into their self-concept (14). In general, individuals evaluate the value and status of their groups through intergroup comparisons and tend to maintain positive social identities. For MCAs, evaluations from patients and their families, acceptance by healthcare teams, and organizational arrangements all can convey information on whether their professional group is recognized and valued, thereby influencing their understanding and identification with their professional identity. Therefore, social identity theory provides a theoretical perspective for analyzing the factors influencing the professional identity of MCAs.

Based on these research gaps, this study adopted a qualitative descriptive research design and collected data on the professional experiences of MCAs through semistructured interviews. An inductive thematic analysis approach was used to explore the factors influencing the professional identity of MCAs, and social identity theory was subsequently applied as an interpretive lens after the themes had been developed. This study aimed to present the social interactional and organizational contexts in which the professional identity of MCAs is shaped and to provide references for healthcare institutions to clarify responsibilities of the role; optimize team collaboration; improve training, incentive systems, and support for career development; and promote workforce stability among MCAs.

## 2 Methods

### 2.1 Study design

This study adopted a descriptive qualitative research design to explore the factors influencing the professional identity of MCAs in China through semistructured interviews. This study was conducted in a tertiary hospital in Wuhan, Hubei province, China, and the data were analyzed using inductive thematic analysis. This report was prepared in accordance with the Consolidated Criteria for Reporting Qualitative Research (COREQ) (15).

### 2.2 Ethical considerations

This study was approved by the Ethics Committee of the School of Medicine, Wuhan University of Science and Technology (approval no.: 2025172).

### 2.3 Participants and recruitment

MCAs were recruited from a tertiary hospital in Wuhan, Hubei province, China, using purposive sampling. Potential participants who met the eligibility criteria were initially contacted with the assistance of personnel responsible for MCA management. Eligible participants were personally approached by the first author, who explained the study and invited them to participate. No prior acquaintance or working, managerial, educational, or supervisory relationship existed between the first author and the participants before recruitment. Participation was voluntary, and participants were informed that they could decline participation or withdraw from reporting at any time. The inclusion criteria are given as follows: (1) medical care assistant certification, (2) prior experience as an MCA for ≥3 months, and (3) ability to clearly articulate personal thoughts and experiences. Participants who were unable to complete the interviews during the data collection period because of reasons such as leave, absence from work, or illness were excluded from the study. Among eligible MCAs, participants were purposively selected for invitation to capture diversity in age, gender, educational level, and length of employment as an MCA while also ensuring that they could provide relevant occupational experiences. These characteristics were considered during recruitment so that participants with different demographic and occupational profiles were included. Data collection and preliminary analysis were conducted together. After the 12th interview, the number of newly generated codes decreased substantially. After three additional participants were interviewed, no new codes emerged, and the coding framework remained stable. Therefore, the research team considered that code saturation had been reached (16). Finally, all 15 MCAs who were selected and invited to participate in this study agreed, and no participants declined or withdrew from the study.

### 2.4 Data collection

Data were collected via one-to-one semistructured interviews. The first author performed data collection from August 2025 to September 2025. All members of the research team had received training in qualitative research and were familiar with interview techniques and data analysis methods. The interviews were conducted by a female postgraduate nursing student. The interviewer was a registered nurse with experience as a clinical nurse; had received qualitative interview training; and was familiar with the research objectives, interview guide, and implementation procedures. The nursing background of the interviewer facilitated the understanding of the clinical context and the work experiences of MCAs. However, although the interviewer had no prior working, managerial, educational, or supervisory relationship with the participants and did not know them before recruitment, her professional identity as a registered nurse may still have influenced the interview interactions. In particular, participants may have been more cautious when describing difficult or negative experiences involving nurses. To minimize this potential influence, the interviewer used open-ended and non-judgmental questions, avoided expressing evaluative opinions during the interviews, and the research team explicitly reflected on the interviewer's professional positionality during data analysis and regular discussions. The corresponding author, who was the supervisor of the interviewer's postgraduate study, provided support for the rigorous implementation of the study and the credibility of the findings. Before the interviews, the researcher informed participants of the purpose and duration of the interview, the procedures for anonymization, and their right to withdraw voluntarily. All the participants signed informed consent forms prior to participation. The main queries that were included in the interview guide are given in the following. (1) Could you please describe your experience working as a medical care assistant and your routine work responsibilities? (2) How do you perceive the profession of medical care assistants, and what do you think are the meanings and values of this work? (3) Please share your experiences and feelings regarding interactions with patients, their families, healthcare professionals, and administrators during your work. (4) Please share the most memorable events or experiences during your work and describe the feelings and reflections they brought to you. (5) Please describe your experiences related to training, learning, or career development during your employment. (6) How do you perceive your current work environment, management practices, and relevant institutional arrangements? (7) During your work as a medical care assistant, what factors influence your perceptions and feelings toward this profession? (8) Are there any other important aspects that have not been mentioned but that you would like to share with us?

All interviews were conducted in a quiet office with only the interviewer and the participant present. During the interviews, the interviewer simultaneously observed and recorded the non-verbal behavior of participants, such as facial expressions and body movements. The average duration of each interview was approximately 30 min. After each interview, the audio recordings were transcribed into textual data, resulting in a total of more than 45,000 Chinese characters of valid transcripts.

### 2.5 Data analysis

An inductive thematic analysis approach was used to analyze the data to explore the factors influencing the professional identity of MCAs in China. Coding and theme development were performed without a predetermined theoretical framework. After the themes had been developed from the interview data, SIT was used to assist in interpreting relevant findings, particularly those concerning social categorization, intergroup comparison, group status, and belonging. Thematic analysis is a qualitative research method that is used to identify and analyze themes or patterns within qualitative data (17). The first author transcribed the interview recordings verbatim into written transcripts, and the second author reviewed the transcripts to ensure their accuracy and completeness. The interview data were analyzed in Chinese. Representative quotations selected for publication were translated into English by the first author and independently checked by the second author for semantic consistency with the original Chinese transcripts. No formal back-translation procedure was performed.

The thematic analysis process in this study followed the six phases proposed by Braun and Clarke (17): (1) familiarizing oneself with the data, (2) generating initial codes, (3) searching for potential themes, (4) reviewing themes, (5) defining and naming final themes, and (6) producing the research report.

First, the first and third authors repeatedly listened to the recordings and read the complete transcripts to become familiar with the details of the interviews with MCAs and to develop preliminary analytical ideas. In the second phase, the first and third authors independently coded the transcripts using NVivo 12 and generated an initial list of codes. The first and third authors subsequently developed a visual map in which codes with similar meanings were compared and grouped into preliminary categories. These categories were then examined for conceptual coherence and organized into candidate subthemes and broader themes. All authors reviewed the identified themes and provided feedback. Candidate themes were retained when they captured a coherent pattern of meaning relevant to the research question, were supported by sufficient data, and could be clearly distinguished from other themes. In the fourth phase, the first and third authors reviewed the coherence and distinctions among candidate themes, discarded themes with insufficient supporting evidence or overlapping content, and returned to the original data for verification.

### 2.6 Trustworthiness of qualitative data

To ensure the rigor and credibility of the study, the qualitative data were systematically evaluated based on four criteria: dependability, credibility, transferability, and confirmability (18). Throughout the analytical process, the research team held regular meetings to review the coding process, theme development, and analytical interpretations. Independent coding and crossreview by the first and third authors, together with team discussions on analytical decisions, were performed to enhance consistency, reflexivity, and transparency. Disagreements that could not be resolved between the two researchers were discussed with the corresponding author. Three participants who were available and willing to continue participating were invited to take part in member checking. They were provided with the theme names, representative quotations associated with each theme, and a summary of the analytical findings and were asked whether these interpretations adequately reflected their experiences and perspectives. The participants considered the themes and interpretations to be consistent with their experiences, and no substantive changes to the analytical framework were required. In addition, detailed records were maintained throughout the processes of data collection, analysis, coding, and theme generation, and these processes were subject to supervision and team review. Finally, the researchers have provided detailed descriptions of the research context, participant characteristics, and study procedures to allow the readers to assess the transferability of the findings to other contexts.

## 3 Results

### 3.1 Participant characteristics

A total of 15 MCAs participated in the interviews and were assigned identification numbers ranging from N1 to N15. The participants were aged between 37 and 58 years, with work experience as MCAs ranging from 0.5 to 3 years. They showed variations in gender, age, marital status, educational level, and work experience. The demographic characteristics of the participants are presented in Table 1. As shown in Figure 1, a word cloud was generated using the word frequency function in NVivo 12.0 based on the original Chinese interview transcripts. Common Chinese stop words and other non-analytically meaningful high-frequency terms were removed before the analysis. The identified high-frequency Chinese terms were subsequently translated into English for presentation in the word cloud while also preserving their meanings in the original interview context. The findings were organized into four overarching themes and eight subthemes. The 33 categories shown in Table 2 were used to organize related coded material within this thematic structure rather than being presented as separate findings. The themes were interconnected within accounts from the participants, particularly through links between role positioning and care interactions, external evaluation and emotional adjustment, and the coexistence of practical needs and challenges to professional dignity.

**Table 1:** Demographic characteristics of the interviewed medical care assistants (*n* = 15).

ID	Age (years)	Gender	Educational level	Work experience (years)	Marital status
N1	53	Female	Junior high school	1	Divorced
N2	50	Female	Primary school	1	Married
N3	47	Female	Bachelor’s degree	2	Married
N4	57	Female	Senior high school	2	Married
N5	58	Male	Junior high school	1	Married
N6	50	Female	Senior high school	3	Married
N7	37	Male	Bachelor’s degree	0.5	Married
N8	51	Female	Junior high school	0.5	Married
N9	55	Female	Primary school	3	Married
N10	52	Female	Primary school	1.5	Divorced
N11	46	Female	Junior high school	1	Married
N12	41	Male	Bachelor’s degree	2	Married
N13	55	Male	Junior high school	3	Married
N14	50	Female	Senior high school	1	Married
N15	53	Female	Junior high school	3	Married

All participants had received medical care assistant training and obtained relevant certificates. Work experience refers to the duration of employment as a medical care assistant.

**Figure 1:**
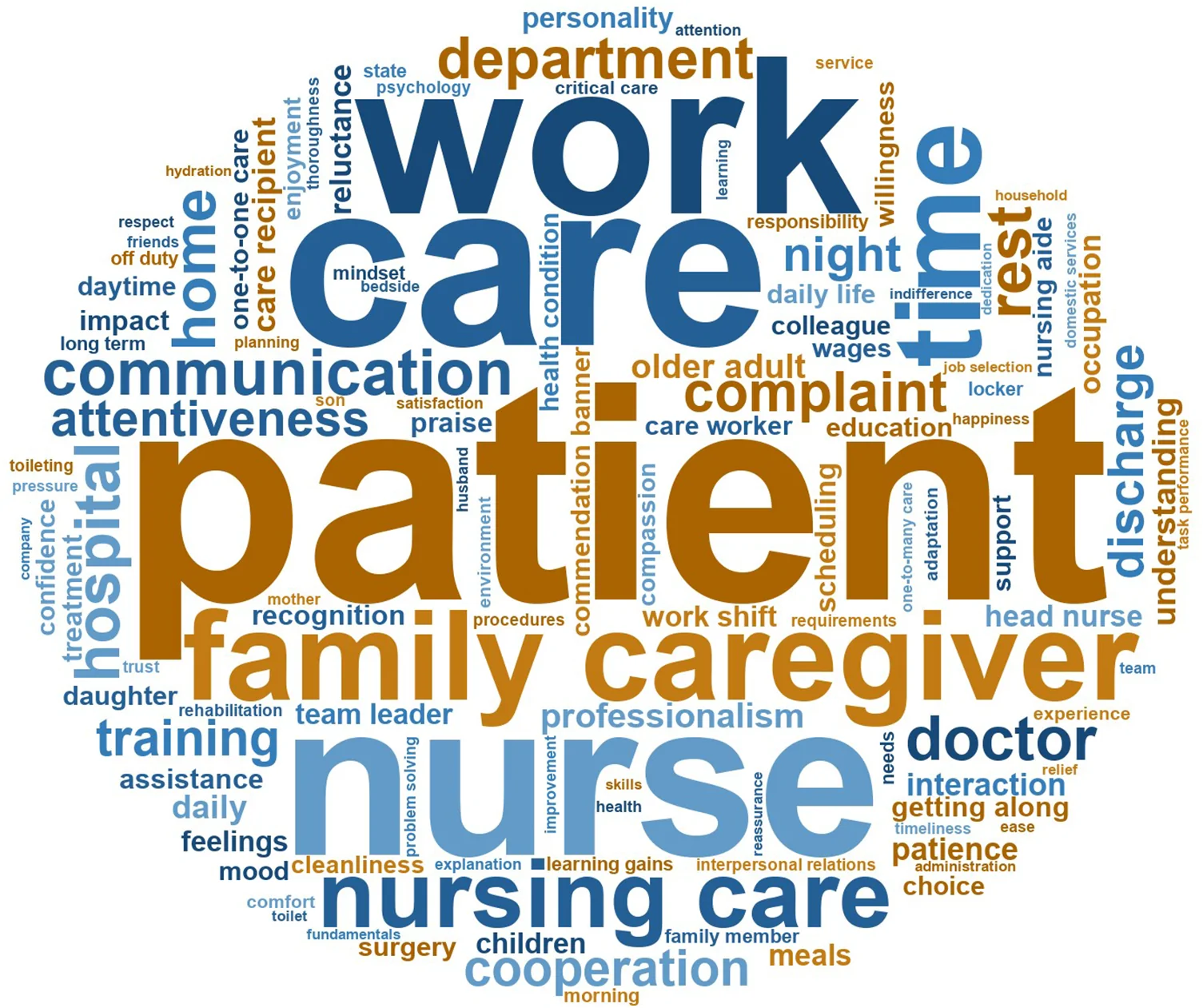
A word cloud of interview transcripts.

**Table 2:** Themes, subthemes, and categories related to factors influencing the professional identity of medical care assistants.

Themes	Subthemes	Categories
Social evaluation influencing experiences of professional value and dignity	Recognition of professional value	Patient appreciation, family recognition, recognition from healthcare professionals, and visibility of work contributions
	Challenges to professional dignity	Experiences of complaints, being misunderstood, being undervalued, and insufficient social recognition
Role positioning and practical needs constructing professional meaning	Professional role positioning	Professional significance of the role, boundaries and responsibilities of the role, assisting healthcare professionals, and meeting care needs
	Practical needs as a driving factor	Social care needs, policy support, family responsibilities, and income and employment needs
Care interactions and self-adjustment shaping professional experiences	Communication and trust building	Active communication, patient trust, patient explanations with patience, and conflict resolution
	Professional competence and emotional adjustment	Professional responsibility, patience and attentiveness, empathy and emotional burden, and perspective-taking and self-regulation
Team and organizational support strengthening professional identity	Acceptance by the healthcare team	Support from head nurses, guidance from nurses, cooperation from healthcare professionals, problem-reporting pathways
	Professional training and institutional support	Training support, certificate recognition, living support, flexible management, performance evaluation and development needs

### 3.2 Social evaluation influencing experiences of professional value and dignity

#### 3.2.1 Recognition of professional value

MCAs frequently interact with patients and their families, and external evaluations serve as an important source for developing their sense of professional value. When their caregiving efforts are recognized and they receive positive feedback, they are more likely to confirm the meaning of their work and their own value, develop a positive perception of their professional group, and subsequently strengthen their professional identity.

I have received two or three appreciation banners, and some patients have even given me flowers. (N5)

One patient brought handmade crafts and home-grown eggs from their family when returning for a follow-up visit. I did not really value these gifts themselves; what I felt more deeply was the recognition and respect they conveyed toward me. (N3)

A family member recognized my attentiveness and sense of responsibility and proactively wrote a letter of appreciation for me. (N11)

#### 3.2.2 Challenges to professional dignity

In contrast to positive evaluations, negative experiences such as complaints, misunderstandings, and being undervalued not only affected the emotional experiences of participants but also weakened their sense of professional dignity. Some participants perceived that the work of MCAs was often undervalued by the public. When their efforts were not understood or recognized, they tended to experience feelings of grievance, frustration, or lack of respect. These negative evaluations led them to perceive that their personal efforts and the value of their professional group were underestimated, which hindered the maintenance of a positive professional identity.

Some patients still looked down on us. (N15)

I was complained about because some people thought that I was afraid of getting dirty and tired. (N9)

Sometimes, when we put in a lot of effort but are not understood, we inevitably feel somewhat aggrieved. (N8)

### 3.3 Role positioning and practical needs constructing professional meaning

#### 3.3.1 Professional role positioning

Professional role positioning reflected how participants understood the scope, responsibilities, and distinctive value of the MCA role in relation to other healthcare professionals. Participants emphasized their roles in daily care, basic nursing support, and psychological support for patients. A clear professional role positioning helped MCAs develop a clearer understanding of their professional identity and their place within the healthcare team.

Our areas of involvement are different. They focus on medical care, whereas we focus more on daily care and basic nursing support, assisting healthcare professionals in their work. (N11)

We are closer to patients than physicians and nurses, and patients tend to trust us more. (N2)

Psychological support for patients still relies on us. (N7)

The connection between role positioning and care interactions was evident from the account of N2. In addition to describing MCAs as closer to patients than physicians and nurses, N2 explained that providing explanations before care procedures helped establish trust in patients. This connected the perceived distinctiveness of the MCA role with everyday communication practices.

#### 3.3.2 Practical needs as a driving factor

In addition to professional role positioning, practical needs also influenced the career choices, occupational expectations, and intentions to remain in the profession for MCAs. Some participants held positive expectations regarding career prospects based on population aging, increasing social care needs, and policy support. Other participants chose this occupation due to family responsibilities, income stability, and employment needs. Therefore, perceptions of role value contributed to the professional meaning that participants attached to their work, whereas practical needs primarily provided tangible motivations for occupational choice and continued employment. These considerations shaped their occupational expectations and intentions to remain in the profession and formed part of the broader context in which professional identity was experienced.

With population aging becoming increasingly high, many older adults require care, resulting in a growing demand for this occupation. (N3)

It is a promising occupation, and national policies also provide considerable support. (N7)

As we are getting older and still need to support our families, we chose this occupation. (N8)

Practical needs also coexisted with challenges to professional dignity. N8 described family responsibilities as a reason for choosing the occupation while also reporting feeling aggrieved when caregiving efforts were not understood. This illustrates how pragmatic reasons for occupational choice could coexist with difficult experiences of professional recognition.

### 3.4 Care interactions and self-adjustment shaping professional experiences

#### 3.4.1 Communication and trust building

Care practices serve as an important context in which MCAs develop and reinforce their professional identity. During continuous and close care interactions, MCAs enhanced understanding and trust in patients and in their families through active communication, patient explanations, and sincere interactions. These interactions helped reduce misunderstandings, improve cooperation in patients, and facilitate the smooth delivery of care.

I communicate with patients before performing any procedures. They are more likely to understand and cooperate; otherwise, they may feel resistant. (N12)

My strength is that I treat everyone with sincerity. (N4)

Before each procedure, I always provide explanations in advance. Once patients understand, their trust is established, and their compliance tends to be higher. (N2)

#### 3.4.2 Professional competence and emotional adjustment

When facing continuous, repetitive, and emotionally demanding care tasks, MCAs perceived dedication, patience, attentiveness, responsibility, and empathy as essential professional qualities required for providing care. Empathy helped them understand the needs of patients, but it could also bring a certain degree of emotional burden. Therefore, when facing changes in the conditions of patients, misunderstandings from family members, and negative emotions, MCAs need to maintain a stable working state through perspective-taking and self-regulation. These professional qualities and adjustment strategies helped them maintain stable caregiving practices and develop positive perceptions of their professional responsibilities and role competence.

Since this is the work I have chosen, I need to have empathy and sincerely help every person suffering from illness. (N1)

This job requires patience, compassion, attentiveness, and a strong sense of responsibility. (N6)

When I see patients with severe conditions, I feel deeply saddened because they are like my own family members. (N5)

Sometimes, when family members misunderstand me, I remind myself not to take these emotions personally. Instead, I try to view the situation from their perspective and show more care and concern. I believe that doing so makes these situations easier to handle. (N3)

External evaluation and emotional adjustment were closely connected in these care encounters. N3 described appreciation from patients as a source of recognition and respect while also using perspective-taking when encountering misunderstanding from family members. These accounts show how responses from patients and families were linked to both experiences of professional value and efforts to manage difficult care interactions.

### 3.5 Team and organizational support strengthening professional identity

#### 3.5.1 Acceptance by the healthcare team

MCAs provide care under the guidance and collaboration of healthcare professionals. Participants reported that physicians, nurses, and head nurses provided them with professional guidance and assistance in addressing work-related difficulties. When the conditions of patients changed, MCAs actively participated in team collaboration by reporting situations promptly and cooperating with subsequent management. Positive collaborative relationships with healthcare professionals and clear reporting pathways facilitated care coordination and supported the sense of acceptance and belonging within the healthcare team among MCAs.

Healthcare professionals and MCAs have a harmonious working relationship. When patients experience problems, healthcare professionals patiently provide explanations, making their work proceed more smoothly. (N3)

The head nurse is very supportive of our team and has helped us resolve many difficulties encountered in our daily work. (N6)

For example, when a patient develops a high fever, I promptly inform the nurse. The nurse then reports the situation to the physician, and I assist with physical cooling measures as needed, such as preparing warm water for wiping the patient or applying ice packs. (N14)

#### 3.5.2 Professional training and institutional support

Professional training and institutional support reflected organizational-level resources and arrangements related to capability development, occupational recognition, and continued employment. Participants reported that on-the-job training helped them acquire caregiving skills, enhance safety awareness, and develop a sense of role competence. The training content included not only basic caregiving skills but also aspects related to patient safety and prevention and control of infections. Some participants further indicated that improvements in professional competence and the acquisition of certificates were associated with greater professional confidence and stronger recognition of the standardization and professionalism of the occupation.

The hospital frequently organizes training sessions, including cardiopulmonary resuscitation, urinary catheter care, sputum clearance, chest percussion, waste classification, and the seven-step handwashing technique. (N6)

Patient safety must always be prioritized, such as the fall prevention measures frequently emphasized during hospital training. (N8)

Obtaining a certificate issued by the National Health Commission also made me feel more confident. (N13)

Beyond professional skills training, institutional support such as accommodation arrangements, leave policies, departmental resource allocation, and performance incentives also influenced the work experiences and occupational stability of MCAs. Human-centered management helped reduce professional and personal stress in MCAs, but further improvements are still needed in performance incentives.

We have dormitories because we need to work rotating shifts. The head nurse also arranged lockers in the department for us to store personal belongings conveniently. (N14)

We only need to pay for water and electricity in the dormitory. If there are important matters, we can request leave in advance, and we are not required to work forcibly. (N4)

I suggest introducing an assessment mechanism and providing performance-based incentives in addition to the basic salary. (N7)

Team and organizational support were also connected with role positioning and care practices. The account of N14 reporting a fever in patient to the nurse and assisting with subsequent care illustrated how team collaboration made responsibilities of the role concrete in everyday practice. Training described by N6 and N8 provided practical guidance for performing these responsibilities, while N13 associated certification with greater confidence. Accommodation and flexible leave arrangements addressed practical needs alongside these forms of professional support.

## 4 Discussion

Social identity theory was used to interpret how social evaluation, role positioning, and team acceptance shaped professional value and sense of belonging among MCAs. The findings further showed that care interactions and organizational support contributed to professional identity, while practical needs and self-adjustment shaped the broader occupational context in which it was experienced. Rather than functioning as isolated factors, these influences intersected within everyday occupational experiences of the participants. Social identity theory proposes that individuals understand themselves based on their group membership and evaluations of the value and social status of the groups to which they belong. A sense of group belonging and the value meanings attached to the group constitute important foundations of social identity (14). For MCAs, recognition from patients and families, acceptance by healthcare teams, clear role boundaries, and organizational support all influenced their understanding and identification with the professional identity of being an MCA.

### 4.1 Social evaluation shaping professional value recognition

Recognition, appreciation, and positive feedback from patients and their families helped enhance perceptions of professional value in MCAs. In contrast, complaints, misunderstandings, and experiences of being undervalued may lead to feelings of grievance, frustration, and impaired professional dignity. Previous studies have shown that evaluations from patients and their families influence job satisfaction and professional experiences among caregivers (19). Long-term or frequent negative feedback may further increase psychological burden, reduce work engagement, and elevate the risk for burnout (4), which are broadly consistent with the findings of the present study.

Social identity theory research suggests that the perceptions of individuals regarding how their occupational groups are evaluated by others influence their judgments of group value and social status, thereby shaping their professional identity (20). The social recognition and professional acceptance of MCAs still require further improvement. Positive evaluations convey messages that the value of their profession is acknowledged, whereas being perceived merely as ordinary caregivers or having their caregiving efforts persistently misunderstood may lead to experiences of undervalued professional worth and marginalized professional identity. Nurses have established relatively well-developed systems of professional titles, career development, and academic evaluation (21–23), and their professional identity is often gradually constructed through professional socialization and integration into the professional group. In contrast, the career development pathways and professional support systems for MCAs remain relatively underdeveloped, making their professional identity potentially more susceptible to daily care interactions and external evaluations. Therefore, hospitals and relevant management institutions should establish standardized mechanisms for positive feedback, complaint communication, and emotional support to promptly address service-related conflicts and maintain the professional dignity of MCAs.

### 4.2 Role positioning and practical needs shaping professional identity

This study found that clear professional role positioning helped MCAs recognize their unique value in daily care, basic nursing support, and psychological support. Previous studies have indicated that unclear role boundaries may reduce perceptions of occupational value, whereas greater role clarity can enhance a sense of responsibility and professional identity (24). This study showed that, by distinguishing their responsibilities from those of physicians and nurses, MCAs gradually clarified their roles in continuous companionship, daily care, and psychological support. From the perspective of social identity theory, this role differentiation can be understood as a form of intergroup comparison through which MCAs clarified the boundaries and relative position of their occupational group within the healthcare team. This process may shape how they understand the value, status, and membership associated with being an MCA (21). When job titles are inconsistent, role boundaries are unclear, or work responsibilities are expanded arbitrarily, MCAs may have difficulty developing a clear professional position. Meanwhile, income security, family responsibilities, and employment needs represented pragmatic motivations for entering and remaining in the occupation. Population aging, increasing social care needs, and policy support also shaped the expectations of participants regarding the future of the profession. These factors are not, in themselves, social identity processes. Instead, they constitute the practical and occupational context in which MCAs evaluate whether their profession is sustainable and worth continuing. In combination with perceptions of role value and professional recognition, such considerations may indirectly shape how participants experience their professional identity. Managers should clarify role boundaries through job descriptions, responsibility lists, and standardized training while improving performance-based incentive mechanisms (25).

### 4.3 Care interactions and self-adjustment strengthening professional identity

In the context of continuous care, communication, empathy, and a sense of responsibility are important conditions for MCAs to provide care and develop positive professional experiences. In this study, patience, attentiveness, and a sense of responsibility helped MCAs complete continuous and repetitive care tasks, while active communication and patient explanations helped reduce misunderstandings and establish trust. By fulfilling caregiving responsibilities conscientiously and receiving understanding and trust from patients and their families, MCAs described greater awareness of their role value, work effectiveness, and competence, which was closely associated with more positive professional identity experiences. Previous studies have also indicated that effective communication, empathy, and a sense of responsibility contribute to the improvement of care relationships and service experiences (26, 27).

Positive feedback obtained through care interactions can further reinforce their perceptions of themselves as members of the professional group and their understanding of the value of that group (23). When MCAs demonstrate patience, attentiveness, responsibility, and empathy in continuous care and gain understanding and trust from patients and their families through effective communication, their positive perceptions as members of the professional group can be strengthened. This finding is broadly consistent with the study of Cao et al. (28), which found that positive emotional feedback, such as appreciation from patients, could enhance the sense of value and job satisfaction among healthcare workers. The significance of care interactions lies not only in obtaining external recognition but also in enabling MCAs to actively establish trust through their own professional behaviors and confirm their self-worth in practice. Accordingly, care interactions were treated primarily as a relational context in which participants received feedback about their caregiving competence and role value; this feedback, rather than interaction quality itself, was interpreted as influencing the professional identity.

Empathy helps MCAs understand illness experiences and actual needs of patients; however, continuously encountering changes in the condition of patients, misunderstandings from family members, and negative emotions may also increase their emotional burden. Perspective-taking and self-regulation help them maintain stable caregiving practices and reduce the adverse effects of negative interactions on their perceptions of professional value and intentions to remain in the profession. Accordingly, self-adjustment was interpreted as a coping process through which participants managed emotional strain and limited the adverse effects of difficult interactions on the perceived professional value. Therefore, it was treated as a process supporting professional identity rather than as a manifestation or outcome of professional identity. Healthcare institutions should provide systematic and continuous training focusing on communication skills, humanistic care, emotional adjustment, and professional responsibility. They should also establish necessary communication and emotional support mechanisms to improve the caregiving capabilities and promote positive professional experiences and professional identity among MCAs.

### 4.4 Team and organizational support consolidating professional identity

This study found that acceptance and collaboration within the healthcare team were important foundations for MCAs to develop a sense of professional belonging. When head nurses and nurses provided work guidance and explanations regarding problems while establishing clear pathways for reporting patient conditions and team collaboration, MCAs were more likely to clarify the responsibilities of their role and perceive the value of their membership as part of the healthcare team. In this sense, acceptance by nurses and other healthcare professionals not only facilitated collaboration but also signaled that MCAs were recognized as legitimate members of the clinical team. Consistent with previous research findings, clear division of responsibilities, effective communication, and team support contribute to the strengthening of care coordination and promotion of the orderly delivery of care (29–31). From the perspective of social identity theory, group belonging and the acceptance and respect experienced by members within the group are important sources for developing positive identity (14, 32). The professional identity of MCAs is not defined solely by their job titles but also depends on whether they are perceived by healthcare teams as trustworthy and valuable team members. Therefore, managers should include MCAs in routine departmental communication, training, and collaborative arrangements while also improving stable mechanisms for work guidance, problem reporting, and team collaboration to enhance their sense of team belonging and professional identity.

Beyond team acceptance, organizational-level institutional support is also an important condition for consolidating the professional identity of MCAs. Social identity theory suggests that organizational arrangements can convey information on whether a professional group is valued and whether its members are recognized as valuable, thereby influencing their professional belonging and professional identity (14). This study showed that standardized training helped improve role competence and patient safety awareness among MCAs, while a professional certification enhanced their professional confidence and recognition of occupational standardization and professionalism. Therefore, professional confidence was interpreted as a competence-related experience arising from these organizational supports; it could support professional identity by affirming perceived role competence and occupational professionalism, but it was not treated as a professional identity itself or as an independent influencing factor. Support such as accommodation, rest arrangements, leave policies, and flexible management helped alleviate practical work pressures, whereas performance-based incentives represented an area requiring further improvement. These findings suggest that organizational support may support the professional identity of MCAs through capability enhancement, supportive employment arrangements, and recognition of professional value. Hospitals and relevant employing institutions should strengthen training, assessment, and performance incentive mechanisms and continue to improve the working environments and humanistic support to promote professional identity and workforce stability.

## 5 Limitations

This study has several limitations. First, purposive sampling was used, and only 15 MCAs from a tertiary grade A hospital in Wuhan were included. In addition, all participants had received occupational training and obtained relevant certificates, and their years of work experience were relatively concentrated.

Given that participants had relatively short lengths of employment as MCAs, the findings may primarily reflect professional identity experiences during the early stage of occupational entry rather than across the full career trajectory. Participants were aged 37–58 years, and therefore, the findings provide limited insight into the experiences of younger MCAs. In addition, these findings reflect the specific institutional context of a large tertiary hospital and may not fully represent the experiences of MCAs working in smaller healthcare institutions, underserved settings, or other employment arrangements. Future studies could expand participant sources and adopt maximum variation sampling to include MCAs of different ages and with diverse geographic backgrounds, institutional settings, lengths of occupational experience, and employment arrangements. Such research could explore similarities and differences across these contexts and help assess the transferability of the findings. Second, the data in this study were mainly obtained through one-time semistructured interviews, and retrospective accounts of the participants may have been affected by recall bias and social desirability bias. Future studies could combine repeated interviews, field observations, and multiple perspectives for data triangulation to further enhance the credibility and comprehensiveness of the findings.

## 6 Conclusion

Professional identity among MCAs is not determined solely by individual motivation or financial compensation but is gradually developed and stabilized through the combined influences of social evaluation, professional role positioning, care practice experiences, and team and organizational support. Positive evaluations from patients, families, and healthcare professionals can enhance the perceptions of professional value among MCAs, whereas misunderstandings, devaluation, and negative feedback may undermine their sense of professional dignity. Clear role boundaries, unique occupational value, and practical employment needs jointly shape professional meaning among MCAs. Communication, empathy, professional responsibility, and emotional adjustment contribute to positive professional experiences and strengthen the professional identity of MCAs. Acceptance by healthcare teams, standardized training, professional certification, and resource support help enhance the sense of professional belonging and occupational stability among MCAs. Therefore, hospitals and relevant employing institutions should clarify responsibilities of the role and boundaries of collaboration, improve mechanisms for positive feedback and complaint communication; integrate MCAs into departmental team development; and strengthen training and certification, psychological support, supportive employment arrangements, and performance-based incentive mechanisms to support professional identity, workforce stability, and care service quality.

## Data Availability

The datasets presented in this article are not readily available because of ethical restrictions and concerns regarding confidentiality of participant information. Requests to access the datasets should be directed to Dongping Xu (email: 2376883853@qq.com).
